# A clinical rule for the difficulty prediction on scalp intravenous access in infants (SIAI) from emergency room

**DOI:** 10.1038/s41598-020-63771-5

**Published:** 2020-04-20

**Authors:** Fengqin Wei, Weiyu Chen, Xiaoti Lin

**Affiliations:** 1 Department of Emergency, Fujian Provincial 2nd People’s Hospital, Affiliated Hospital of Fujian University of Traditional Chinese Medicine, Fuzhou, China; 2 Department of Physiology, Zhongshan Medical School, Sun Yat-sen University, Guangzhou, China; 3 Department of Breast, Fujian Provincial Maternity and Children’s Hospital of Fujian Medical University, Fuzhou, China; 4 Department of Breast Oncology, State Key Laboratory of Oncology in South China, Sun Yat-sen University Cancer Center, Collaborative Innovation Center for Cancer Medicine, Guangzhou, China

**Keywords:** Health care, Medical research, Risk factors

## Abstract

Infant intravenous access poses a significant challenge to the operator. Scalp vein is the ideal location for emergency medical staff to perform intravenous access for administration of fluids or medications. To tackle this challenge, we developed a clinical rule for the difficulty prediction on scalp intravenous access in infants (SIAI) conducting a prospective cohort study in a pediatric emergency room. A total of 658 infant patients who underwent SVI from January 2017 to September 2018 were recruited in this study. The failure rate of SIAI on the first attempt was 20.2%. Five variables, including dehydration condition, obesity, vein invisibility, vein impalpability and hyperactive status of infant, were independently and statistically associated with failure rate of SIAI. Furthermore, we indicated that any one alone of the above five variables did not significantly lead to greater than 50% failure rate of indwelling needle SIAI (*p* > 0.05). However, summary effects of more than one of these five variables were statistically significant associated with greater than 50% failure rate of SIAI (*p* < 0.05). When employing the five-variable model, validation cohort subjects displayed dehydration, obesity, vein invisibility, vein impalpability and hyperactive status had a 67.5% likelihood of failed first attempt on SIAI (C = 0.675; 95% CI: 0.622–0.727; *p* < 0.001). For the first time, we developed the difficult model for SIAI. We found that dehydration, obesity, vein invisibility, vein impalpability and hyperactive status of the infant patients are the independent and significant predictors associated with SIAI failure. Our predicted model indicates that infant patients with combination of more than one of the five variables contribute to greater than 50% failure rate of indwelling needle in SIAI.

## Introduction

Intravenous access for sampling of blood or administration of medications is widely used in pediatric emergency department^1,2^. However, infant intravenous access poses a significant challenge to the operators, such as nurses and doctors. Failure of intravenous access not only aggravates pain and dissatisfaction of patients, but also enlarges the burden of the operators. Thus, it is a great benefit for emergency medical staff to reduce the failure rate of infant venous puncture.

In order to predict the difficulty of peripheral intravenous access in children, Yen *et al*. previously developed a difficult peripheral intravenous access score^3^. Furthermore, they found that it is more difficult to perform peripheral intravenous access on infants, compared with children older than 1 year^3^. Similarly, Riker *et al*. observed that there existed high failure rate for operator to establish intravenous access in infants^4^. However, both of these two studies did not specify the locations of peripheral venous access, such as veins of upper limb, lower limb, external jugular or scalp. Therefore, the difficulty model of intravenous access on a specific location of infants remains to be developed.

Most of the infant inpatients have weak constitution and are prone to serious complications, leading to adverse events. Therefore, it is significant that the emergency medical staffs to determine an ideal venous access to perform the treatment administration to the infant patients in the shortest possible time frame. Overall, locations of intravenous placement include peripheral venous access and central venous access. However, central venous access is not the first option and rarely used in pediatric emergency department, as the complications caused by central venous access can place massive physical burden on children patients^1^. On the other hand, most operators prefer to choose the superficial veins of upper limb or lower limb as the first attempt in clinical practice. Locations of peripheral intravenous placement mainly include superficial veins of upper limb (*e.g*. hand back vein and antebrachium vein), lower limb (*e.g*. dorsocuboidal vein and medial malleolus vein), external jugular and scalp. Nevertheless, it is very common that peripheral veins of upper limb and lower limb in infant patients are not completely developed, which increases the difficulty of peripheral intravenous access on both upper limb and lower limb in infants, owing to small and deeply-located veins^5^. As the neck of infant patients is relatively flexible and difficult to stable, the external jugular vein is not a reliable site for infant intravenous access^6^. However, the appearances of vein visibility and vein palpability are relative clear in scalp venous of infants. Therefore, it is an optimal location for emergency medical staff to choose scalp vein for venous access in infants.

With the goal of tackling the challenge of scalp intravenous access in infants (SIAI), we tried to develop a clinically predictive rule for SIAI. Preliminarily, we performed a prospective feasibility study to explore the candidate variables for the failure rate of scalp venous indwelling needle insertion in infants. We further sought to determine the potential independent variables for failed venous insertion on the first attempt. Ultimately, we aimed to develop a SIAI difficult degree model that is simple to apply and useful for the perdition of the failure rate of indwelling needle intravenous access in infants.

## Results

### Study characteristics

**Figure 1:**
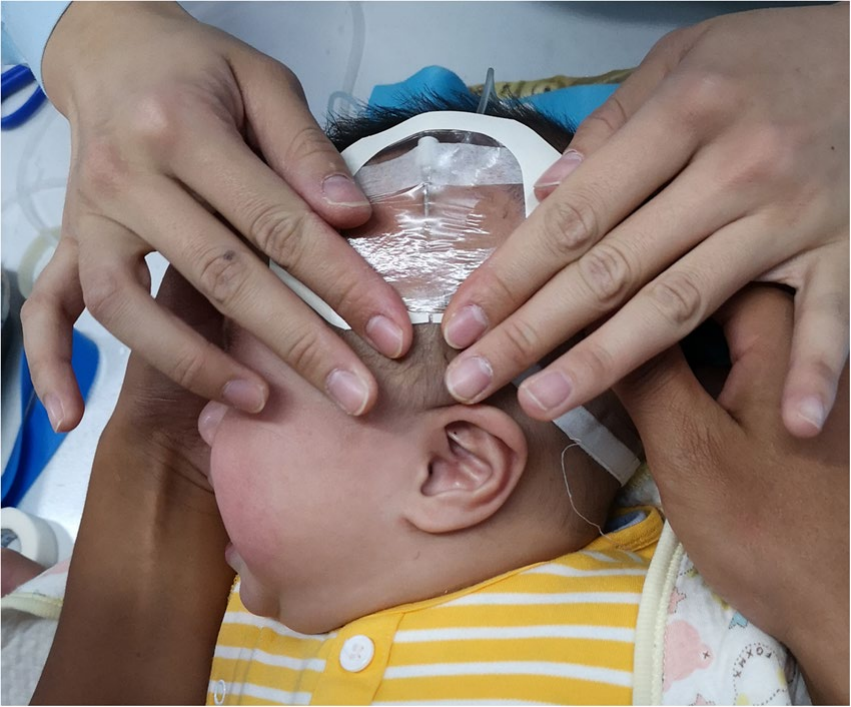
Representative image of scalp indwelling needle in SIAI.

A total of 658 infants were enrolled. The male-female ratio was 1:1.1. The age range was 0 to 12 months. Of the subjects, 11.1% were younger than 4 weeks (neonate), 42.9% were aged from 4 weeks to 6 months, and 46.0% were aged from 6 months to 1 year. Representative image of scalp indwelling needle intravenous access in infants was presented in Fig. 1. All together, the failure rate of SIAI on the first attempt was 20.2%.

### Univariate correlation analyses of the association between predictor variables and failure rate of first attempt on SIAI

Initially, in order to reveal the candidate variables associated with failure rate in SIAI placement, we included candidate variables of both patient and operator. As shown in Table 1, twenty-two candidate predictor variables were involved into the univariate analysis. We observed that ten of the twenty-two variables were statistically associated with the failure rate on first SIAI attempt. The detailed significant variables including mass, dehydration condition, obesity, location of intravenous attempt, vein visibility, vein palpability, calm status of infant, prematurity history, reason for intravenous attempt and nursing prediction of difficulty level (*p* < 0.05).

**Table 1:** Univariate analysis of the association between predictor variables and outcome of first intravenous attempt in SIAI.

Variables	First intravenous attempt (n)		*x*^2^	*p* value
	Success	Failure		
Totality	525	133		
Age			2.425	0.297
≤4 weeks (neonate)	61	12		
4 weeks~6 months	230	52		
6~12 months	234	69		
Sex			0.021	0.885
Male	245	63		
Female	280	70		
Mass			10.329	0.035*
≤3 Kg	83	33		
3~5Kg	141	26		
5~8Kg	120	38		
8~10Kg	104	21		
>10Kg	77	15		
Height			5.392	0.145
≤50 cm	97	33		
50~60 cm	135	40		
60~70 cm	147	28		
>70 cm	146	32		
Dehydration condition			10.554	0.001*
Presence	47	25		
Absence	478	108		
Obesity			8.490	0.004*
Presence	66	30		
Absence	459	103		
Interval time of last intravenous access			0.835	0.659
≤2 weeks	178	49		
2 weeks~6 months	171	38		
>6 months	176	46		
Sterilization method			0.179	0.915
Alcohol	188	47		
Iodine	168	45		
Others	169	41		
Parent inside therapy room			2.18	0.140
Presence	503	131		
Absence	22	2		
Local anesthetics			0.550	0.458
Presence	89	19		
Absence	436	114		
Totality	525	133		
Location of intravenous attempt			6.225	0.044*
Superficial temporal vein	181	52		
Vein of forehead	282	57		
Posterior auricular vein	62	24		
Vein visibility			6.523	0.011*
Clear	444	100		
Unclear	81	33		
Vein palpability			4.804	0.028*
Presence	269	54		
Absence	256	79		
Drawing marker before intravenous access			0.018	0.893
Presence	198	51		
Absence	327	82		
Calm status of infant			5.530	0.019*
Calm	434	98		
Hyperactive	91	35		
Prematurity history			3.960	0.047*
Presence	64	25		
Absence	461	108		
Reason for intravenous attempt			6.177	0.046*
Medication only	188	53		
Fluids only	213	39		
Both medications and fluids	124	41		
Parental prediction of cooperativeness			3.314	0.069
Satisfied	291	62		
Unsatisfied	234	71		
Parental prediction of difficulty level			2.740	0.098
Difficult	246	73		
Easy	279	60		
Nursing prediction of difficulty level			5.207	0.022*
Difficult	185	33		
Easy	340	100		
Nursing prediction of cooperativeness			2.721	0.099
Satisfied	435	118		
Unsatisfied	90	15		
Self-confidence of nursing			1.684	0.194
Presence	263	75		
Absence	262	58		

*p < 0.05.

### Multivariate logistic regression of independent and significant predictor variables associated with the failure rate of SIAI

To determine the independent and significant predictor variables, we further performed a multivariate logistic regression analysis to evaluate the statistically significant factors in univariate analysis. All the ten significant variables were appraised by a multivariate logistic regression model in a backward stepwise regression screening. In all significant predictor variables, we found that the following five variables, including body mass, location of intravenous access, prematurity history, reason for intravenous attempt and nursing prediction of difficulty level, were weakly predictive. Finally, the other five strongly predictive variables were retained in the predicted model: dehydration condition, obesity, vein visibility, vein palpability, and calm status of infant (*p* < 0.05; Table 2).

**Table 2:** Binary logistic regression multivariate analytic results of 5 independent and significant variables from 658 cases with indwelling needle SIAI.

Variables	Coefficient	Standard deviation	Wald	*p* value	HR	95% CI
Dehydration condition (presence)	−0.894	0.280	10.230	0.001	0.409	0.236–0.707
Obesity (presence)	−0.790	0.257	9.448	0.002	0.454	0.274–0.751
Vein visibility (clear)	1.207	0.293	16.902	<0.001	3.342	1.880–5.941
Vein palpability (presence)	0.906	0.249	13.245	<0.001	2.474	1.519–4.030
Calm status of infant (calm)	0.673	0.239	7.917	0.005	1.960	1.227–3.132

*p < 0.05.

### Development of the difficulty model of SIAI on both the receiver operating characteristic (ROC) curve and the area under the curve (AUC), as well as its summary index

To validate the difficulty of SIAI, we performed the multiple-variable model to test their summary effects of ROC curve and the AUC (Table 3). In regard to one-variable model, we found that anyone of the five variables alone does not significantly associated with more than 50% failure rate of indwelling needle SIAI (*p* > 0.05). On the contrary, all results of the two-variable model, three-variable model, four-variable model and five-variable model indicated that their summary effects are statistically significant related to more than 50% SIAI failure rate (*p* < 0.05).

**Table 3:** Predictive values of each SIAI difficult degree in multiple-variable model.

Multiple-variable model		Sensitivity	Specificity	Youden	Area (95% CI)	*p* value
One-variable	Dehydration condition (presence) alone, (D)	0.910	0.188	0.098	0.549 (0.492–0.606)	0.079
	Obesity (presence) alone, (O)	0.874	0.226	0.100	0.550 (0.493–0.607)	0.075
	Vein visibility (unclear) alone, (VV)	0.846	0.248	0.094	0.547 (0.490–0.603)	0.094
	Vein palpability (absence) alone, (VP)	0.512	0.594	0.106	0.553 (0.497–0.608)	0.058
	Calm status of infant (hyperactive) alone, (C)	0.827	0.263	0.090	0.545 (0.489–0.601)	0.109
Two-variable	D plus O	—	—	—	0.585 (0.528–0.642)	0.002*
	D plus VV	—	—	—	0.588 (0.532–0.645)	0.002*
	D plus VP	—	—	—	0.585 (0.530–0.641)	0.002*
	D plus C	—	—	—	0.578 (0.521–0.635)	0.005*
	O plus VV	—	—	—	0.580 (0.524–0.637)	0.004*
	O plus VP	—	—	—	0.582 (0.526–0.637)	0.004*
	O plus C	—	—	—	0.583 (0.527–0.639)	0.003*
	VV plus VP	—	—	—	0.615 (0.564–0.666)	<0.001*
	VV plus C	—	—	—	0.569 (0.512–0.625)	0.014*
	VP plus C	—	—	—	0.581 (0.527–0.636)	0.004*
Three-variable	—	—	—	—	>0.5	<0.05*
Four-variable	—	—	—	—	>0.5	<0.05*
Five-variable	Total	—	—	—	0.675 (0.622–0.727)	<0.001*

^*^p < 0.05.

**Figure 2:**
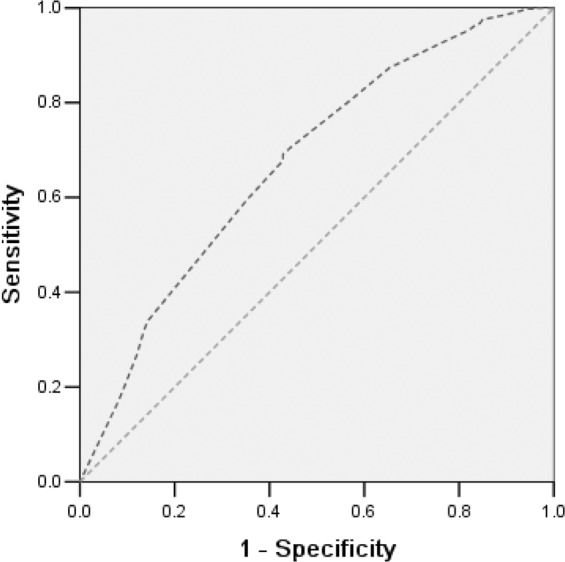
Receiver operating characteristic (ROC) curves of 5-variable proportionally weighted rule for the difficult prediction on SIAI.

When employing the five-variable model, the overall C-statistic for the multinomial model was C = 0.675 (95%CI: 0.622–0.727; *p* < 0.001). So, validation cohort subjects with dehydration, obesity, vein invisibility, vein impalpability and hyperactive status of infant patients had a 67.5% of failed first attempt on SIAI (AUC = 0.675; Fig. 2).

## Discussion

To the best of our knowledge, for the first time, our current study evaluated the difficult degree of indwelling needle insertion in SIAI. Moreover, our prospective feasible cohort study developed the SIAI difficult model. We found that the following five variables, including dehydration condition, obesity, vein visibility, vein palpability, and calm status of infant, were the independent and significant predictors for the failure on the first attempt of SIAI. As far as these five independent and significant predictors were concerned, both variables of patient and operator were involved. Among these five variables, dehydration condition and obesity are the constitution of the infant patients. If both of these two variables were presented in infants, the failure rate of SIAI would be higher than 50%. Furthermore, variables of vein visibility, vein palpability and calm status of infant are the factors that depended on capability of the operators for SIAI. Apart from choosing an ideal location for venous access according to vein visibility and palpability of an infant patient, the operator is also suggested to promote infant’s resilience to eliminate the adverse influence induced by stresses^7,8^.

Since the success rate of SIAI on the first attempt in infants was 79.8%, we believed that scalp intravenous access is the optimal option for operator to perform venous access. In clinical practice, an experienced operator is needed if SIAI needs to be performed on an infant displayed more than one of five variables (presence of dehydration condition, presence of obesity, vein invisibility, vein impalpability and hyperactive status of infant). Moreover, it is advised that SIAI attempts should be limited to 3. The emergency medical staff could choose superficial veins of upper limb (*e.g*. hand back vein and antebrachium vein) or lower limb (*e.g*. dorsocuboidal vein and medial malleolus vein) for intravenous access if the above attempts of SIAI are failed. It is also recommended that the attempts of venous access into superficial veins of limbs should be limited to 3. Peripherally inserted central catheters (PICC) maybe the other choice of venous access, if the peripheral venous access was not succeeded^9^. Besides, VeinViewer and ultrasound-assisted approaches should be used for delineating the running course of subcutaneous veins, rather than blind approach in a complicate situation^10^. In addition, peripherally inserted central venous catheters are potential options for intravenous access in infant patients^11^.

The major limitation of the current study is that the enrolled infants were recruited from one hospital. Besides, the sample size of the present study was relatively small. In addition, only medical staffs who worked for the emergency service at least five years was included in this study. Therefore, well-designed researches with large sample sizes are needed to further demonstrate the SIAI difficult model.

In conclusion, for the first time, we developed the difficult model of SIAI, which can be simple to apply and be useful for predicting the failure rate of indwelling needle in SIAI. We found that dehydration condition, obesity, vein invisibility, vein impalpability and calm status of infant are independently and significantly related to failure rate of SIAI. Moreover, the current predicted model indicated that infant patients with combination of two or more of these five variables leads to greater than 50% failure rate of indwelling needle SIAI.

## Methods

### Patient population and data collection

This was a prospective cohort study conducted between January 2017 and September 2018. The emergency room has around 50000 attendances per year, of which approximately 500 are infants. All the experimental methods were carried out in accordance with the ethical standards of the institutional and/or national research committee and with the 1964 Helsinki declaration and its later amendments or comparable ethical standards. The study was approved by the hospital ethics committee of Fujian Provincial 2^nd^ People’s Hospital, Affiliated Hospital of Fujian University of Traditional Chinese Medicine. Written informed consent was obtained from all parents of the infants in this study.

All infants (age range: 0~12 months) requiring venous insertion were eligible for inclusion in this study. Skin shade of all involving participants was yellow. We excluded subjects who required immediate emergency care, puncture point with apparent arteriopalmus or had skin disease on scalp. Both doctors and nurses who had been working for emergency service more than five years were eligible to participate in this study. Before the placement attempt was performed, demographic data of the study were recorded. Candidate predictor variables included the following twenty-two items: age, sex, mass, height, dehydration condition, obesity, interval time of last intravenous access, sterilization method, parent inside therapy room, local anesthesia, location of intravenous attempt, vein visibility, vein palpability, drawing marker before intravenous access, calm status of the infant, prematurity history, reason for intravenous attempt, parental prediction of cooperativeness, parental prediction of difficulty, nursing prediction of difficulty, nursing prediction of cooperativeness and self-confidence of nursing.

Such special outcome measures were described as following. Infant with Kaup (kg/m^2^) index more than 18 was considered as obesity^12^. Vein visibility referred to the nurse’s ability to see the vein before SIAI. Vein palpability was labeled as the nurse’s ability to feel the vein before SIAI. Patients born at less than 38 week-gestation were considered as a positive history of prematurity^13^. In addition, the primary endpoint was defined as failed SIAI on first attempt. Conversely, a successful venous insertion referred to a saline flush could be injected without compromising the vein and ready for use.

### Data analysis and model development

All data were computed using the Statistical Software Package for the social Sciences (SPSS version 13.0, SPSS, USA). Preliminarily, the association between predictor variables and the failure of first intravenous attempt was calculated using Pearson chi square for trends or Fisher’s exact test, depending on the condition of data. A two-tailed *p* value less than 0.05 was considered statistically significant. Following, significant variables of the above univariate analyses were appraised by a multivariate logistic regression model in a backward stepwise regression screening (*p* < 0.1). Statistical significance of the remaining candidate variable was assessed using hazard ratio (HR) with 95% confidence interval (CI). Later, the decision rules were based on the ROC curve and the AUC as its summary index. The concordance (C) statistic was performed to measures discrimination of ROC and AUC (range from 0.5 to 1.0)^14–16^.
